# Spatial Distribution of the Pathways of Cholesterol Homeostasis in Human Retina

**DOI:** 10.1371/journal.pone.0037926

**Published:** 2012-05-22

**Authors:** Wenchao Zheng, Rachel E. Reem, Saida Omarova, Suber Huang, Pier Luigi DiPatre, Casey D. Charvet, Christine A. Curcio, Irina A. Pikuleva

**Affiliations:** 1 Department of Ophthalmology and Visual Sciences, Case Western Reserve University, Cleveland, Ohio, United States of America; 2 University Hospitals, Cleveland, Ohio, United States of America; 3 Department of Pathology, University of Texas Medical Branch, Galveston, Texas, United States of America; 4 Department of Ophthalmology, University of Alabama, Birmingham, Alabama, United States of America; University of Tennessee, United States of America

## Abstract

**Background:**

The retina is a light-sensitive tissue lining the inner surface of the eye and one of the few human organs whose cholesterol maintenance is still poorly understood. Challenges in studies of the retina include its complex multicellular and multilayered structure; unique cell types and functions; and specific physico-chemical environment.

**Methodology/Principal Findings:**

We isolated specimens of the neural retina (NR) and underlying retinal pigment epithelium (RPE)/choroid from six deceased human donors and evaluated them for expression of genes and proteins representing the major pathways of cholesterol input, output and regulation. Eighty-four genes were studied by PCR array, 16 genes were assessed by quantitative real time PCR, and 13 proteins were characterized by immunohistochemistry. Cholesterol distribution among different retinal layers was analyzed as well by histochemical staining with filipin. Our major findings pertain to two adjacent retinal layers: the photoreceptor outer segments of NR and the RPE. We demonstrate that in the photoreceptor outer segments, cholesterol biosynthesis, catabolism and regulation via LXR and SREBP are weak or absent and cholesterol content is the lowest of all retinal layers. Cholesterol maintenance in the RPE is different, yet the gene expression also does not appear to be regulated by the SREBPs and varies significantly among different individuals.

**Conclusions/Significance:**

This comprehensive investigation provides important insights into the relationship and spatial distribution of different pathways of cholesterol input, output and regulation in the NR-RPE region. The data obtained are important for deciphering the putative link between cholesterol and age-related macular degeneration, a major cause of irreversible vision loss in the elderly.

## Introduction

**Figure 1:**
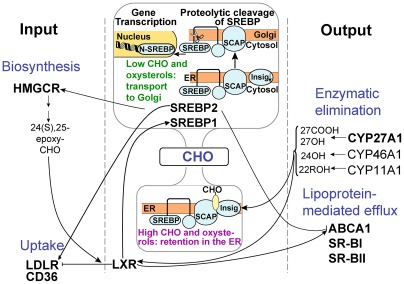
Cholesterol homeostasis. Simplified representation of the coordinate regulation of the pathways of cholesterol (CHO) input and output indicating proteins investigated in the present work. HMGCR, 3-hydroxy-3-methylglutaryl-CoA reductase, the rate limiting enzyme in cholesterol biosynthesis; LDLR and CD36, receptors recognizing low density lipoproteins (LDL); LXR, the liver X receptor, transcription factor suppressing the expression of LDLR and activating the expression of SREBP1 and ABCA1; SREBP, SCAP and Insig, proteins activating the expression of HMGCR and LDLR; CYP27A1, CYP46A1 and CYP11A1, cytochromes P450 that metabolize cholesterol to 5-cholestenoic acid (27COOH), 27-hydroxycholesterol (27OH), 24-hydroxycholesterol (24OH) and 22R-hydroxycholesterol (22ROH), respectively; ABCA1, cholesterol efflux transporter; SR-BI and SR-BII, scavenger receptor SR-BI and its splice variant SR-BII recognizing HDL. Arrows and blunt ends indicate positive and negative regulators, respectively. The dumbbell-shaped object in the middle of the figure shows the SREBP pathways when cholesterol levels are high (bottom compartment) and low (top compartment). SREBPs are synthesized on the endoplasmic reticulum (ER) and form a complex with the escort protein SCAP. When sterol levels are low (top compartment), SCAP transports SREBPs to the Golgi, where the active form of SREBP is generated and initiates the transcription of target genes in the nucleus. When sterol concentrations are high (bottom compartment), cholesterol binds to SCAP triggering its interaction with the ER-resident protein Insig, whereas oxysterols bind to Insig eliciting its complex formation with SCAP. As a result, the SREBP/SCAP/Insig complex is retained in the ER. The cartoon showing the regulation of cholesterol biosynthesis is reproduced/adapted with permission from Meer, G. and Kroon, A. (2011) J. Cell Sci., 124, 5–8 (http://jcs.biologists.org/content/124/1/5.long).

Cholesterol is present in every mammalian cell and is essential for cell growth and viability [1]. Extrahepatic cells acquire cholesterol from endogenous biosynthesis and circulating low density lipoproteins (LDL) and remove cholesterol excess via reverse transport by high density lipoproteins (HDL) and/or metabolism to oxysterols by cytochromes P450 (CYP) 27A1, 46A1 and 11A1 (Fig. 1) [2], [3], [4]. Elaborate mechanisms regulate and link the pathways of cholesterol acquisition and elimination so that cholesterol input equals cholesterol output (reviewed in [5], [6], [7], [8]). Central to the system controlling cholesterol input is a family of proteins called SREBPs (Text S1) which form complexes with the escort protein SCAP. At high cholesterol and oxysterol concentrations the SREBP-SCAP complex is retained in the endoplasmic reticulum (ER) by the ER retention protein Insig. At low sterol levels the SREBP-SCAP complex leaves the ER and SREBPs initiate the transcription of target genes in the nucleus [5]. The SREBP isoform 1a is a potent activator of all SREBP-responsive genes including those that mediate the biosynthesis of cholesterol, fatty acids, and triglycerides, whereas SREBP1c and SREBP2 preferentially act on genes of fatty acid and cholesterol biosynthesis, respectively. [9]. At high expression levels, however, each isoform can activate the biosynthesis of both fatty acids and cholesterol [9]. SREBP1c is transcriptionally regulated by liver X receptor (LXR) [9], which in turn is activated by oxysterols many of which are generated by P450s [7], [10]. LXR also stimulates the transcription of genes involved in cholesterol removal including the efflux transporter ABCA1 and downregulates LDLR, the receptor for LDL [10], [11], [12]. Thus, LXR plays a crucial role in integrating the pathways of cholesterol input and output.

**Figure 2:**
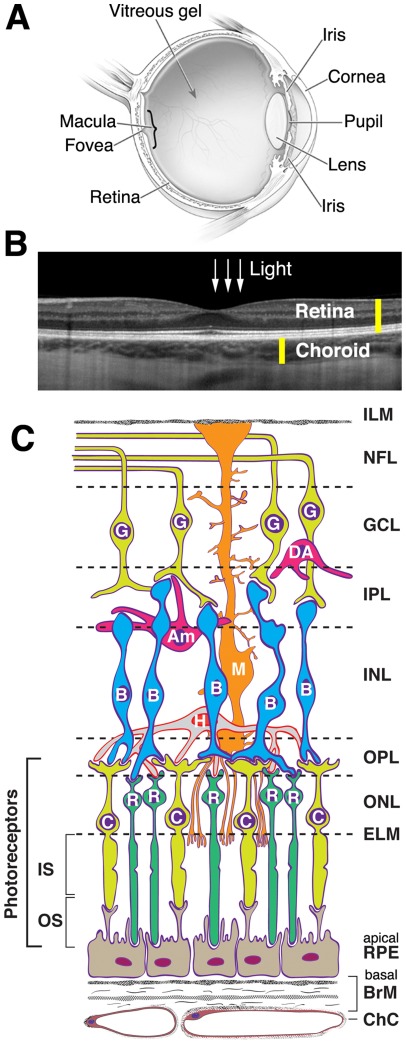
Human Eye. **A,** cross-section of a human eye. The neurosensory retina (central nervous system) and choroid (vascular bed for the photoreceptors and RPE) are part of the inner lining. The macula (a 6 mm diameter area responsible for central vision) and fovea (a depression in the macula) are bracketed. Schematic available at http://www.nei.nih.gov/health/eyediagram/index.asp. **B,** human retina and choroid *in vivo*. Spectral domain optical coherence tomography with enhanced depth imaging. Scan of macula, courtesy of R.F. Spaide, MD. **C,** chorioretinal cells and layers. Cells: RPE, retinal pigment epithelium (nurse cells to the photoreceptors); C, cone photoreceptor; R, rod photoreceptor; H, horizontal cell (interneuron); B, bipolar cell (interneuron); M, Müller cell (radial glial cell); Am, amacrine cell (interneuron); DA, displaced amacrine cell (interneuron); G, ganglion cell (output neuron). Müller cells (M) extend almost the width of the retina; their apical processes form the ELM, and their foot processes partially form the ILM. Layers: ChC, choriocapillaris (capillary bed for RPE and photoreceptors); BrM, Bruch's membrane (vessel wall and RPE substratum); ELM, external limiting membrane (junctional complexes); ONL, outer nuclear layer; OPL, outer plexiform layer (synapses); INL, inner nuclear layer; IPL, inner plexiform layer; GCL, ganglion cell layer; NFL, nerve fiber layer (ganglion cell axons); ILM, inner limiting membrane. Non-photoreceptor layers of the retina are supplied by the retinal circulation (not shown). Graphics by D. Fisher; inspired by Figure 4–2 of Ryan SJ, editor. Retina: Mosby; 2006.

Very little is currently known about the maintenance of cholesterol homeostasis in the retina (Figs. 2A, B), the sensory organ in the back of the eye that converts light energy to electrochemical signals transmitted to the brain through the optic nerve. Embryologically part of the central nervous system, the retina has several layers of neurons interconnected by synapses as well as glial cells (Fig. 2C). Vision is initiated by the light-sensitive rod and cone photoreceptors that form the outer surface of the retina. These are supported by the retinal pigment epithelium (RPE), a polarized monolayer that provides diverse services essential for optimal photoreceptor health. One of these services is supply of nutrients to the photoreceptors from the choroidal circulation located external to the RPE. The choroid has the highest blood flow per unit volume in the body, and is separated from the RPE by Bruch's membrane (BrM). The RPE is unique among epithelia in that it faces neurosensory retina (NR) within the blood-retina-barrier on its apical surface and the systemic circulation through the choroid on its basal surface. Photoreceptors, RPE, BrM, and choroid are the layers primarily impacted by age-related macular degeneration (AMD), an incurable disease that leads to a loss of central vision in affected older adults.

Several features of chorioretinal biology make understanding its cholesterol homeostasis an interesting but difficult task. First, the mechanisms of cholesterol acquisition and elimination likely vary in cells of the NR-RPE region according to cell type and function. While in the NR cholesterol is probably derived from two sources, endogenous biosynthesis and systemic circulation [13], [14], [15], the RPE has a third potential source–membrane-rich photoreceptor outer segments (OS). Although the OS are relatively poor in cholesterol, 10% of them are phagocytosed every day by the RPE [15] and thus could produce high cholesterol load in bulk. Second, unlike many organs that rely on cholesterol removal via nascent HDL synthesized in the liver and intestine, the NR has been proposed to synthesize its own lipoprotein particles (HDL-like) to mediate intra-retinal cholesterol exchange [16]. The importance of this exchange is implicated by evidence for associations between AMD and genes historically associated with plasma HDL metabolism without evidence for a consistent relationship between plasma HDL levels and AMD [17], [18], [19]. Intra-tissue cholesterol exchange along with cholesterol metabolism to oxysterols, also shown to occur in the NR [20], make the NR similar to the brain. Third, besides the HDL-mediated reverse transport, the RPE appears to have an additional mechanism of cholesterol elimination: like the liver, this layer has the capacity for basolateral secretion of lipoprotein particles containing apolipoprotein B (the major protein component of LDL) rich in esterified cholesterol (EC) [21], [22]. These lipoprotein particles accumulate with age in BrM and contribute the largest single component to AMD's hallmark extracellular, lipid-containing lesions (drusen and basal linear deposits) [21], [22]. Finally, the retina has a unique environment (exposure to light, high metabolic rate, and high content of polyunsaturated fatty acids) that contributes to its vulnerability to oxidative stress.

The present work provides first insights into the relationship and spatial distribution of different pathways of cholesterol input, output and regulation in the NR-RPE region. We found that cholesterol maintenance in the OS is significantly different from that in other retinal layers and that the gene regulation in the RPE does not involve the SREBP mechanism. Our studies also show significant inter-individual variability in gene expression in the RPE in contrast to the retina.

## Materials and Methods

### Human specimens

Our human tissue use conformed to the Declaration of Helsinki and was approved by the Institutional Review Boards at Case Western Reserve University and University of Texas Medical Branch at Galveston. Eyes were obtained from de-identified human donors from the Cleveland Eye Bank following written informed consent of the respective families. Samples of human brain were obtained also following written informed consent of the respective families. Demographic information on the donors, death-to-preservation time and pertinent medical history are summarized in Table S1. Only eyes with no apparent retinal pathology were used as assessed by examination of post-mortem fundus photographs by a fellowship trained retina-vitreous specialist following initial gross inspection of the posterior pole under the dissecting microscope with 3x magnification. Of each pair, one globe was preserved in 4% paraformaldehyde for histochemistry studies and the companion globe was dissected to obtain a 8-mm trephine punch of the peripheral retina (∼5 mm temporally and parallel to the macula and optic nerve). The trephine punch was first bisected with a razor blade, and one half of each punch was placed under the dissecting microscope. The NR was carefully separated from the underlying RPE-choroid and immediately placed in RNeasy RLT buffer (Qiagen, Germantown, MD). The RPE was carefully scraped from BrM/choroid with a crescent knife (Katena Products, Inc., Denville, NJ) and suspended with several drops of water to facilitate collection with a microcapillary tube and transfer to RNeasy RLT buffer. If a visible tear or blood was observed in the BrM/choroid interface, the RPE above this region was not collected. Eye processing was within 11–16 hrs post-mortem. To evaluate/account for cross-contamination, mRNA isolated from the NR and RPE (see section 2.2) was subjected to quantitative real-time PCR (see section 2.4) for the presence of *ABCA4* specific for NR [23] and *RPE65* highly expressed in the RPE [24]. In the NR, the levels of *RPE65* were very low (<0.5% of the levels in the RPE) and similar in different donors indicating low contamination of NR by the RPE. In the RPE, expression of photoreceptor-specific *ABCA4* was ∼30% of those in the NR. This could be due to RPE contamination from adjacent photoreceptors, phagocytosis of photoreceptors and leaky expression of the *ABCA4* gene. Regardless of the reason, the levels of the *ABCA4* in the RPE were similar in different donors indicating consistency of dissection.

### RNA isolation and cDNA synthesis

Total RNA was isolated using the RNeasy Mini kit (Qiagen, Germantown, MD), and contaminating genomic DNA was completely eliminated by treatment with the RNase-Free DNase Set (Qiagen, Germantown, MD). RNA was considered DNA-free when 40 cycles of real-time PCR did not give an amplification signal with the primers for β-actin (ACTB) (Table S3). One microgram of RNA was utilized for each reverse transcriptase reaction using SuperScript III Reverse Transcriptase (Invitrogen, Carlsbad, CA).

### PCR array

The RT^2^ Profiler “Human Lipoprotein Signaling & Cholesterol Metabolism” PCR array system (SABiosciences, Frederick, MD) was used. The PCR reactions were carried out using the RT^2^ SYBR Green Master Mixes (SABiosciences, Frederick, MD) and an ABI 7000 Sequence Detection System (Applied Biosystems, Foster City, CA). The threshold cycle (Ct) for each gene was identified by the 7000 SDS 1.1 RQ Software (Applied Biosystems, Foster City, CA) with the threshold value and baseline being 0.75 and automatic, respectively, in the analysis settings. ΔCt was then calculated by subtracting the mean Ct of the five housekeeping genes from the individual Ct. The following housekeeping genes were used: beta-2-microglobulin (B2M), hypoxanthine phosphoribosyltransferase 1 (HPRT1), ribosomal protein L13a (RPL13A), glyceraldehyde-3-phosphate dehydrogenase (GAPDH), and ACTB.

### Quantitative real-time PCR (qRT-PCR)

The primers for qRT-PCR (Table S2) were designed to generate small amplicons (<130 bp) to enhance detection sensitivity and reduce bias in degraded tissue. The amplification efficiency of all the primer sets was >90%. For each gene of interest in each sample, expression was measured in triplicate and normalized to the expression of ACTB; SD was <10%. Products from qRT-PCR were isolated by PureLin PCR Purification Kit (Invitrogen, Carlsbad, CA) and sequenced.

### Tissue cryosectioning for immunohistochemistry

Eye globes were kept for 24 hrs in 4% paraformaldehyde/0.1 M potassium phosphate buffer (KP_i_), pH 7.2, and then transferred to 1% paraformaldehyde/0.1 M KP_i_, pH 7.2, and kept at 4°C until dissected. Upon dissection of each eye, the anterior segment was removed, and an 8 mm×4 mm rectangle of the temporal peripheral retina (comprised of the NR, RPE and choroid) was cut with one edge of the rectangle starting at the margin of the *ora serrata* and the other edge ending ∼10 mm from the optic nerve. The perpendicular border of the rectangle originated superiorly and proceeded inferiorly. Tissue rectangles were embedded in OCT (Electron Microscopy Sciences, Hatfield, PA), frozen in liquid nitrogen and cryosectioned at 10 µm. Sections were placed on glass slides, dried at room temperature and stored at −20°C until used.

### Immunohistochemical staining

Frozen retinal sections were warmed to room temperature for 30 min and fixed for 10 min with acetone pre-cooled at −20°C. Following acetone evaporation, sections were washed twice by 5-min incubations with phosphate buffered saline (PBS), treated with the blocking buffer (3% goat serum containing 2% BSA in PBS) at room temperature for 30 min, and left overnight at 4°C in the blocking buffer containing primary antibodies (Abs). Next morning, sections were rinsed three times with PBS and incubated at room temperature for 45 min with secondary Abs. Slides were then washed with distilled water and incubated at room temperature for 10 min with a solution of 0.035% (W/V) Sudan black in 70% ethanol to reduce autofluorescence [25]. Following washes with distilled water, sections were covered by Prolong Gold antifade mounting media containing DAPI (Invitrogen Corporation, Carlsbad, CA) and protected with a coverslip. The primary Abs used for immunostainings are described in Table S3. The secondary Abs were Dylight 649-labeled goat anti-rabbit and donkey anti-goat IgG (Jackson ImmunoReserach Laboratories, Inc., West Grove, PA) diluted 1∶150. Stained slides were imaged on a Leica DMI 6000 B inverted microscope (Leica Microsystems Wetzlar, Germany) using a Retiga EXI camera (Q-imaging Vancouver British Columbia). Image analysis was performed using Metamorph Imaging Software (Molecular Devices Downington, PA). Secondary Abs were visualized by excitation at 652 nm and collection of emissions at 670 nm, whereas the excitation and emission wavelengths for the DAPI detection were 350 nm and 460 nm, respectively. All images were taken with matched exposure times for experimental and control sections.

### Filipin staining

This was carried out as described [26]. Previous studies also validated the use of filipin for histochemistry staining by parallel results with enzymatic, chromatographic and mass spectrometry assays (reviewed in [27]). Sections were removed from the freezer, air-dried for 1 hr, and rehydrated with PBS three times for 5 min. To detect unesterified cholesterol (UC), filipin III (Cayman Chemical, Ann Arbor, MI), 50 µg/ml in PBS prepared from a 3.3 mg/ml stock in dimethylsulfoxide, was applied to slides for 1 hr in a light-blocking box. Slides were then rinsed three times with PBS and coverslipped with the Vectsashield mounting medium containing propidium iodide (Vector Laboratories, Inc., Burlingame, CA). Detection of EC required two additional steps prior to filipin treatment: extraction of UC with 70% ethanol for 30 min, and hydrolysis of EC by cholesterol esterase (Sigma-Aldrich, 15 µg/ml in 0.1 M KP_i_, pH 7.2) for 3.5 hrs at 37°C followed by the three 5-min washes with PBS. Filipin fluorescence was excited at 340–380 nm and emission collected at 385–470 nm. The excitation and emission wavelengths for the propidium iodide detection were 535 nm and 615 nm, respectively. Exposure time of experimental and control images for UC was 15 msec and those for EC was 400 msec.

## Results

### Profiling of gene expression by PCR array

**Figure 3:**
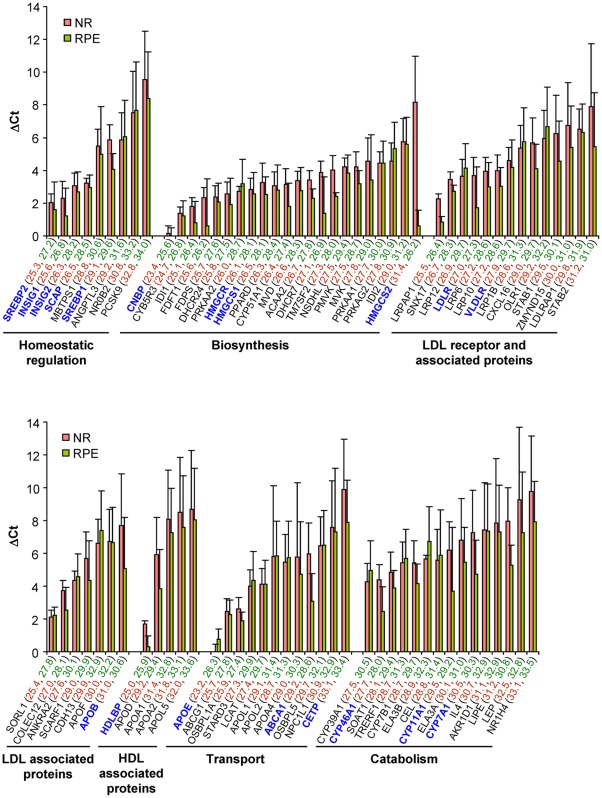
Profiling of gene expression by PCR array. Genes for 84 proteins involved in maintenance of cholesterol homeostasis were evaluated in the NR (pink bars) and RPE (green bars). Each bar represents the mean ΔCt ± SD of the independent measurements in 6 donors. Individual Ct values are shown in parenthesis, the color code is the same as for the bars. Genes mentioned in the Results section are shown in bold and colored in blue.

Gene expression was assessed in 6 donors (Fig. 3) and involved the analysis of 84 genes from the major pathways of cholesterol maintenance: biosynthesis and uptake of cholesterol from systemic circulation; intracellular cholesterol processing, trafficking, storage and regulation; and cholesterol elimination via metabolism and lipoproteins. In all donors every gene in the array was detected in both NR and RPE, yet at a different PCR Ct value, which, in general, reflects the level of gene expression (lower Ct corresponds to the higher gene expression; accordingly, lower ΔCt also corresponds to the higher gene expression since it represents normalized expression relative to the mean of the five housekeeping genes). In the NR, the two most abundant genes were APOE (cholesterol transport) and CNBP (cholesterol biosynthesis), whose average Ct values (23.2 and 23.4, respectively) were comparable to those of some of the five housekeeping genes: GAPDH, ACTB, RPL13A, HPRT1, and B2M (19.2, 22.1, 24.0, 25.5, and 25.6, respectively). Hence, ΔCt values of APOE and CNBP were very low (0 and 0.16, respectively). CNBP was also one of the two most abundant genes in the RPE (Ct/ΔCt = 25.6/0) along with HDLBP (HDL associated proteins) having Ct/ΔCt equal to 25.9/0.3. For comparison, the Ct values of the five housekeeping genes in the RPE were 21.8 (GADPH), 24.4 (ACTB), 26.0 (RPL13A), 29.0 (HPRT1), and 26.6 (B2M). In general, genes in the NR and RPE were detected at comparable ΔCt values with the only exception being HMGCS2 (cholesterol biosynthesis) expressed preferentially in the RPE. With respect to the function, in both NR and RPE, genes related to cholesterol biosynthesis were detected at lower ΔCt values than genes from other groups suggesting that endogenous biosynthesis in an important contributor to the total pool of cholesterol in the NR and RPE.

PCR is a very sensitive technique and detects low abundance genes that are not always translated into protein. The PCR array detected the gene for the liver-specific enzyme CYP7A1 (cholesterol catabolism), but we could not confirm protein expression of CYP7A1 either in the NR or RPE even with the most sensitive mass spectrometry technique, multiple reaction monitoring (I. Pikuleva and I. Turko, unpublished observations). Conversely, ΔCt values of apoB (LDL associated proteins) and CETP (cholesterol transport), shown to be expressed as proteins in the RPE (apoB) and NR (CETP) by other methods [16], [21], were at levels above those of CYP7A1. If, nevertheless, to use the ΔCt of CYP7A1 (7.3 and 4.7 in the NR and RPE, respectively) as an arbitrary borderline value above which gene expression should be interpreted with extreme caution, still 63 genes in the NR and 45 genes in RPE are below this value and thus have a potential to also be expressed as proteins.

### Quantification of gene expression by qRT-PCR

To confirm the results of the PCR array, relative qRT-PCR was used. Three groups of genes were selected for evaluation: genes pertinent to the SREBP (SREBPs 1 and 2, SCAP, Insigs 1 and 2, LXRs α and β, HMGCR, LDLR and ABCA1); genes responsible for enzymatic cholesterol removal (CYPs 27A1, 46A1, and 11A1); and genes encoding scavenger receptors involved in reverse cholesterol transport (SR-BI, SR-BII, and CD36). The latter three as well as CYP27A1 and LXRs α and β, were not encompassed by the PCR array and, therefore, evaluated in the previous section. Yet, these six proteins are known to be present in the NR and RPE as shown by immunohistochemistry (IH) and mass spectrometry, and suggested by qRT-PCR [28], [29], [30], [31], [32].

**Figure 4:**
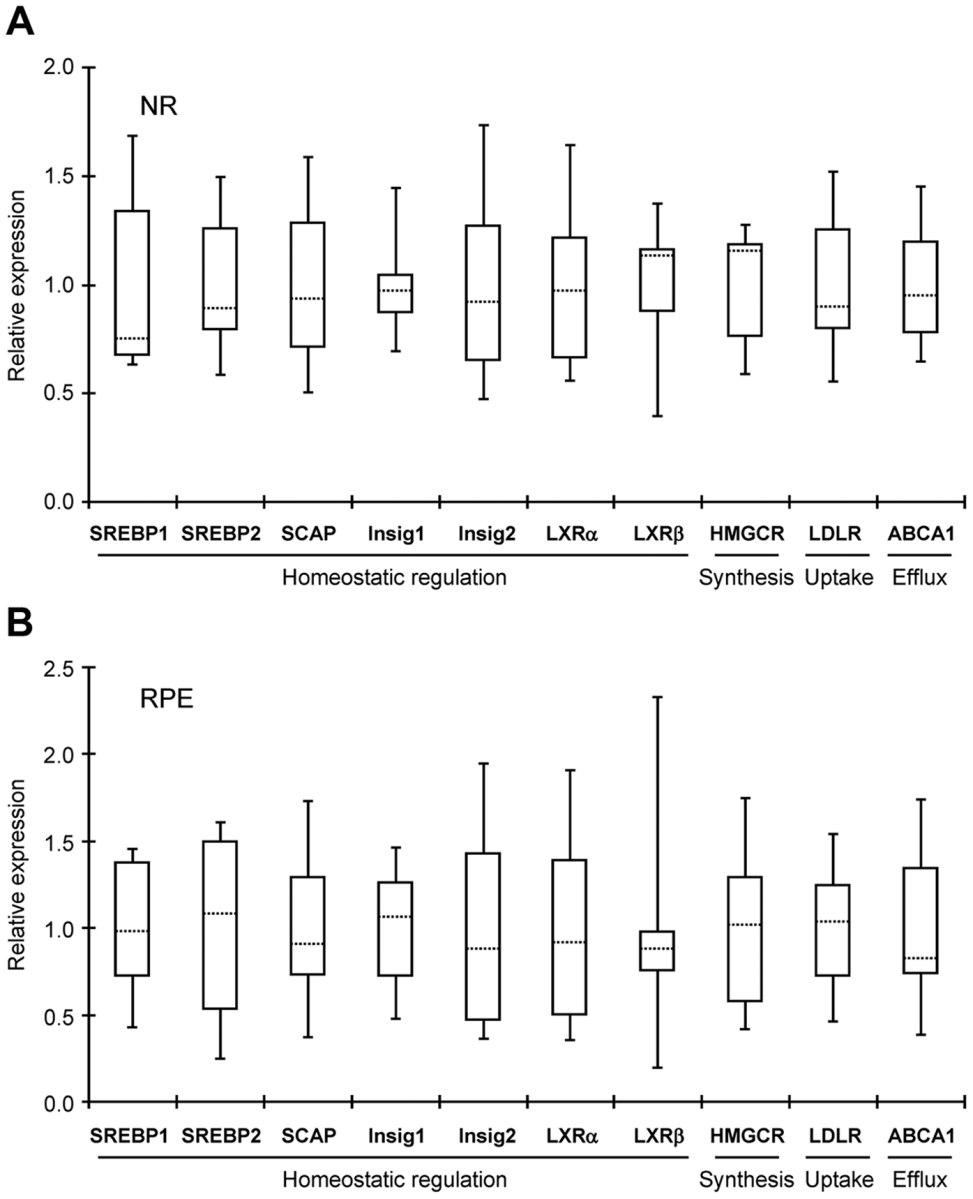
Gene expression as assessed by qRT-PCR. **A**, NR. **B**, RPE. Key proteins involved in homeostatic regulation, synthesis, uptake and efflux of cholesterol were evaluated. Their gene expression was measured and normalized based on the expression of ACTB in the same sample. For each gene, the mean of the gene expression in 6 donors was then calculated and assigned a value of “1” on the Y-axis. The gene expression in the individual sample was then compared to this mean value giving a number of relative gene expression on Y-axis. Data are presented in the form of Whisker-box plots in which the box area encompasses middle 50% of expression level values, the dotted line represents the sample median and the whiskers represent upper 25% (top whisker) and lower 25% (bottom whisker) of expression level values.

All 16 genes selected for assessment by qRT-PCR were found to be expressed in the NR and RPE of all six donors analyzed previously by the PCR array, although at levels varying between the donors. Inter-donor variability in the expression of genes from the first group (SREBP1, SREBP2, SCAP, Insig 1, Insig 2, HMGCR, LDLR, and ABCA1) was small (2.1–3.7-fold) in the NR and moderate (3.1–6.6-fold) in the RPE (Fig. 4). The only exception was LXRβ whose expression varied up to 12.3-fold in the RPE.

**Figure 5 pone-0037926-g005:**
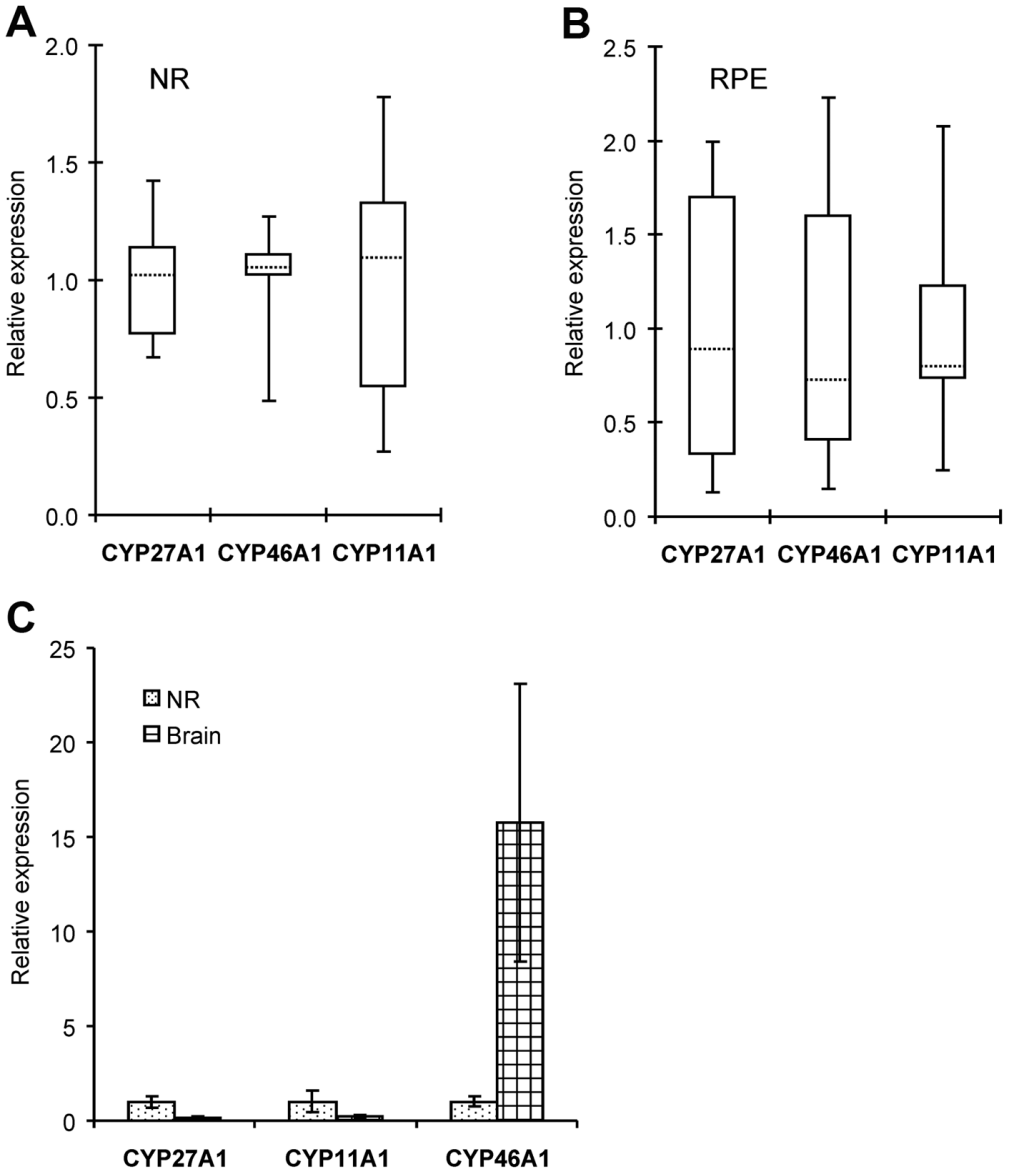
Gene expression of cholesterol-catabolizing P450s as assessed by qRT-PCR. **A,** NR. **B**, RPE. **C**, brain. In **A** and **B,** data presentation is the same as in Fig. 4; in **C,** each bar represents the mean ± SD of the independent measurements in 6 donors of the retina and 4 donors of the brain. The latter are not the same as donors of the retina. Information on brain donors could be found in ref. 56. In all panels, gene normalization is as in Fig. 4.

Similarly, the levels of mRNA transcripts for CYP27A1 and CYP46A1 varied only a little (2.1–2.6-fold) between the donors in the NR and significantly (15.5–15.7-fold) in the RPE (Fig. 5). This is in contrast to CYP11A1, whose variations in gene expression were similar in both NR and RPE (up to 6.7- and 8.6-fold, respectively). The amounts of mRNA for cholesterol-catabolizing P450s were also measured in the brain, which similar to the retina is a part of the central nervous system. In gray matter of the temporal lobe, the gene expression of CYPs 27A1 and 11A1 was on average 7.7- and 4.5-times lower than in the NR, whereas that of CYP46A1 was 15.8-fold higher (Fig. 5). Inter-individual variability in the brain was small, up to 3-fold for CYP27A1 and CYP46A1 and up to 2-fold for CYP11A1.

**Figure 6:**
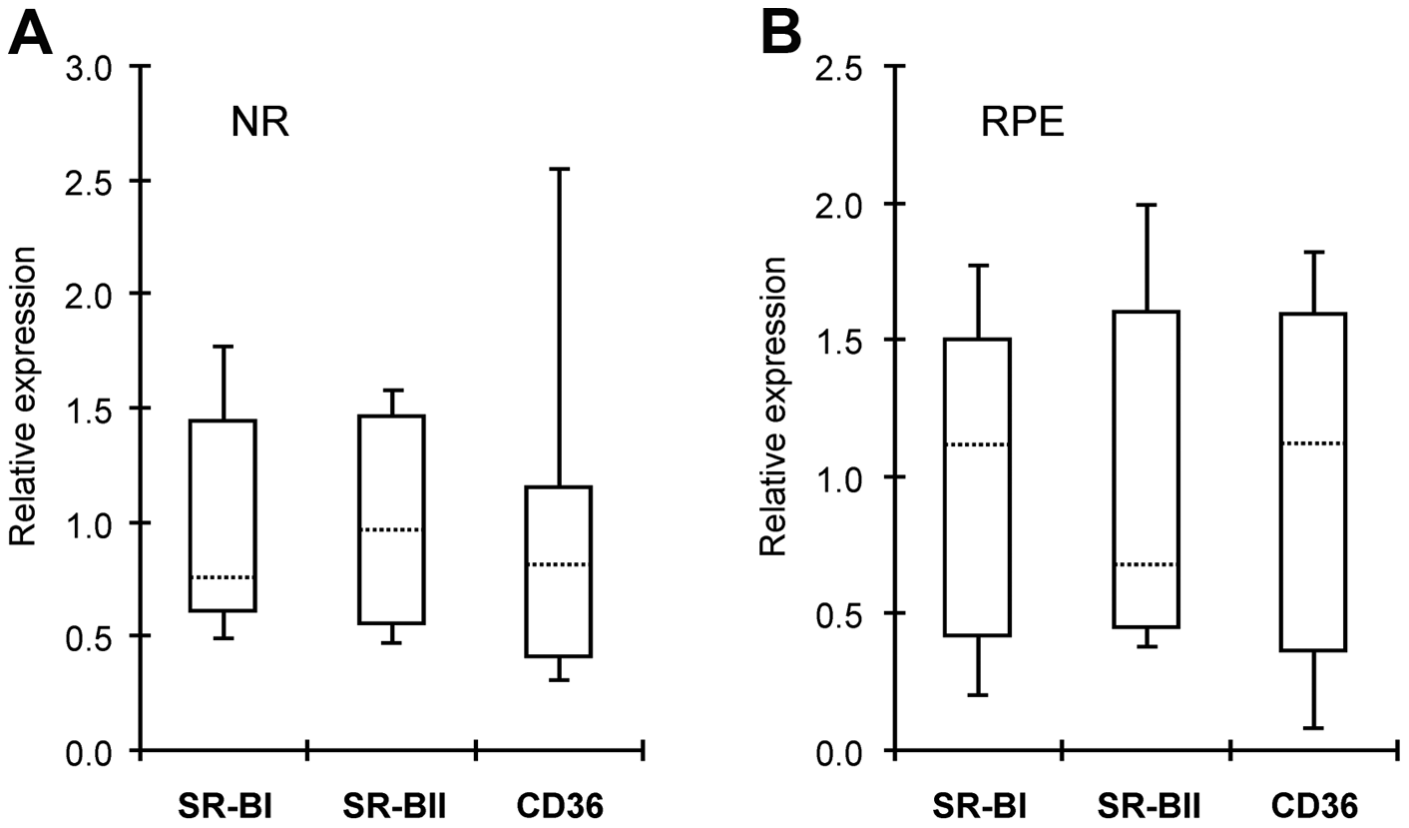
Gene expression of scavenger receptors as assessed by qRT-PCR. **A,** NR. **B**, RPE. Donors, gene normalization and data presentation are the same as in Fig. 4.

Finally, expression of the genes from the third group (SR-BI, SR-BII and CD36) varied moderately (3.7–8.5-fold) in the NR and significantly (9–23-fold) in the RPE (Fig. 6) with CD36 showing the highest inter-individual variability among all the genes quantified by qRT-PCR in both NR (8.5-fold) and RPE (23.1-fold).

### Retinal localization of the proteins by IH

Grossly normal peripheral retinas from 6 different donors were used for studies by IH. Structurally, peripheral retina is very similar to macula, yet is thinner and more highly dominated by rods (rod:cone ratio is ∼25∶1 for periphery, and 9∶1 for macula [33]). Peripheral retina also lacks the Henle fiber layer formed by extended processes of foveal photoreceptors and Müller cells. For consistency, immunofluorescent images are shown for the retina from one donor (PM023), in which the largest number of proteins could be demonstrated. Patterns of immunostaining in this donor were also observed in at least 2 other donors. Two groups of proteins were analyzed: proteins from the SREBP-mediated pathways (SREBPs 1 and 2, SCAP, Insigs 1 and 2, LXRs α and β, HMGCR, LDLR and ABCA1) and cholesterol-catabolizing P450s (CYPs 27A1, 11A1, and 46A1).

**Figure 7:**
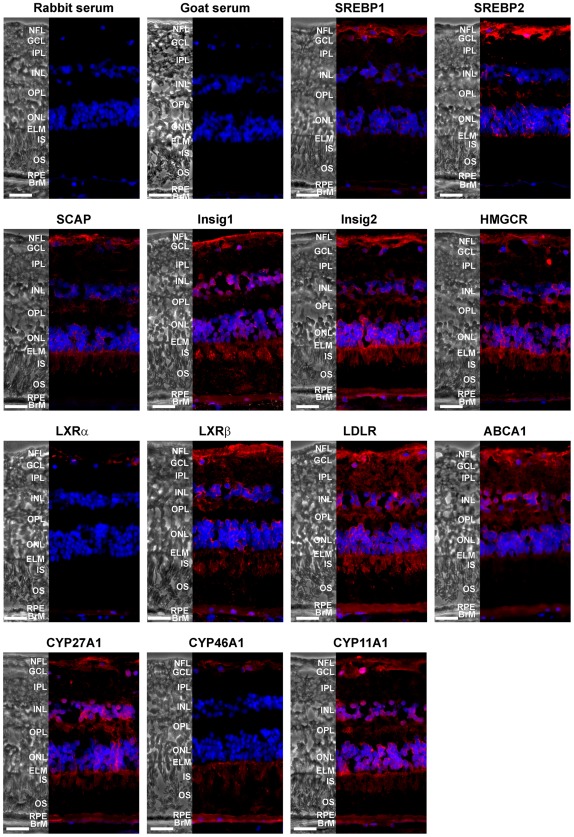
Protein Expression. IH localizations of proteins involved in regulation of cholesterol homeostasis (SREBPs, SCAP, Insigs and LXRs), cholesterol biosynthesis (HMGCR), uptake (LDLR), efflux (ABCA1) and catabolism (CYPs 27A1, 46A1, and 11A1) in the retina of donor PM023. Phase contrast images (on the left of each panel) are given for comparison. Nuclei were stained by DAPI (blue) and immunoreactivity was detected by DyLight 649 conjugated secondary Abs (red). Staining with serum from non-immunized animal (rabbit or goat) served as a negative control. Scale bars are equal to 30 µm. Abbreviations of retinal layers are the same as in Fig. 2.

Proteins of the SREBP/SCAP/Insig complex appear to co-localize in the nerve fiber layer (NFL) and three nuclear layers–the ganglion cell layer (GCL), inner nuclear layer (INL) and outer nuclear layer (ONL) (Fig. 7). Immunoreactivity for SREBP2, SCAP and Insigs was also observed in the two synaptic layers – the outer plexiform layer (OPL) and external limiting membrane (ELM). Photoreceptor inner segments (IS) showed strong staining only for SCAP and Insigs 1 and 2, whereas the OS had signal only for Insig 1. Insigs were also detected in the RPE and BrM. Thus, expression of SREBPs, SCAP and Insigs does not overlap in all retinal layers: only cell bodies of retinal neurons and axons of the ganglion cells seem to express all three proteins suggesting that at these locations cholesterol biosynthesis is strongly controlled at transcriptional level.

HMGCR and LDLR are among the multiple proteins in the pathways of cholesterol input that are regulated by SREBP [5], [9], [34]. Immunoreactivity for HMGCR and LDLR was detected in the same retinal layers where the proteins of the SREBP/SCAP/Insig complex co-localize: NFL, GCL, INL and ONL (Fig. 7). In addition, HMGCR and LDLR were also immunostained in the IPL, OPL, ELM, IS, and RPE. Immunolocalization of HMGCR and LDLR in human retina showed a more expanded pattern of expression than rat and monkey retinas, respectively [13], [14]. In rats, strong immunoreactivity for HMGCR was localized only to Muller cells, IS and RPE [13]. In monkeys, considerable immunostaining for LDLR was observed only in GCL, OPL, RPE and choriocapillaries with faint staining in IS. In general, the discrepancy in staining patterns could be due to interspecies variations and different source of primary Abs. We, however, used Abs for LDLR from the same vendor as in studies on monkeys at a similar dilution (1∶150 in our work *vs* 1∶100 in ref. [14]). Further work is required to determine the basis of this discrepancy. Despite the differences, neither previous [13], [14] nor our studies detected expression of HMGCR and LDLR in the OS.

Expression of SREBP-1c is controlled by LXRs [9]. Immunoreactivity for LXRα, known to be primarily expressed in the liver, kidney and macrophages [35], was very faint in human NR and localized only to NFL (Fig. 7). In contrast, staining for ubiquitous LXRβ was more pronounced and included NFL/GCL, IPL, INL, OPL, ONL, ELM, IS, RPE and BrM. Thus, LXRβ and SREBP1 showed co-localization only in the NF/GCL and INL, whereas in other layers (IPL, INL, OPL, ONL, ELM, RPE and BrM) expression of LXRβ coincided with that of ABCA1 and LDLR regulated by this transcription factor [10], [11], [12]. ABCA1 also seems to be co-localized with SREBP2 (NF/GCL, INL, OPL, ONL, and ELM), which negatively regulates ABCA1 [36], [37]. Thus, not only the pathways of cholesterol input but also of output are well controlled in several retinal layers in NR. Immunolocalization of ABCA1 in human retina was more similar to that in mouse retina [38], [39] than in monkey retina [16]. This difference occurred despite our using the same vendor Abs, although at a different dilution (1∶1,000 in this study vs. to 1∶200 and 1∶500 in ref. [38], [39] and [16], respectively). While further work is required to understand these differences, in all four studies signal was not observed in the OS.

LXRs are activated, at least *in vitro*, by oxysterols (27-hydroxycholesterol, 24S-hydroxycholesterol, and 22R-hydroxycholesterol) [40] produced by cholesterol-catabolizing P450s CYP27A1, CYP46A1 and CYP11A1, respectively [41]. Signals for CYP27A1 and CYP11A1 were observed in the same retinal layers as that for LXRβ (NFL/GCL, INL, OPL, ONL, ELM, IS, RPE and BrM); immunostaining for CYP46A1 was seen mainly in the NFL/GCL, IS, RPE and BrM (Fig. 7). However, previous studies on monkeys show that CYP27A1 is mainly localized to the IS with only faint immunostaining in other retinal layers [42]. These differences with our data could be due to different retinal regions used (macula in studies on monkeys and peripheral retina in studies on humans) as well as different quality of Abs. To assess the latter, we performed Western blot analysis of human retinal homogenate with anti-CYP27A1 Abs. Only one band corresponding to the molecular weight of purified CYP27A1 was observed (Fig. S1A), thus confirming the specificity of our Abs. Retinal quantities of less abundant CYP46A1 were below the limits of detection by our anti-CYP46A1 Abs (data not shown), therefore IH was performed on retinas from wild type and CYP46A1 knockout mice (KO) (Fig. S1B). Immunoreactive signal was absent in KO mice but present in wild type animals with a pattern of staining similar to that of previously reported for mice [43]. Yet, this pattern was different from staining of human retina (Fig. 7). Immunolocalizations on mice confirmed the quality of our CYP46A1 Abs and also reveal that there are interspecies differences in retinal localization of CYP46A1 between humans and rodents [43], [44]. With respect to CYP11A1, IH on human retina showed an expanded expression pattern relative to that in rats and hamsters [45], [46]. In both rodent species immunoreactivity was mainly confined to only two retinal layers, the GCL and INL [45], [46].

### Retinal distribution of cholesterol

Retinal sections adjacent to those used for IH were stained with filipin, a fluorescent antibiotic interacting specifically with the free 3β-hydroxyl group of cholesterol and other sterols, thus enabling detection of the unesterified forms of sterols [47]. Cholesterol is the most abundant sterol in the retina, present at concentrations several orders of magnitude higher that those of other sterols [20], [48], [49], therefore filipin fluorescence mainly reflects staining of UC. Filipin can also be used to identify EC in tissues that have been extracted with ethanol and pre-treated with cholesterol esterase.

**Figure 8:**
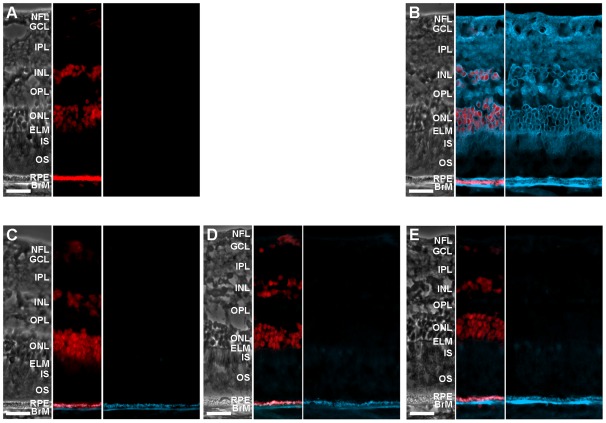
Histochemical detection of UC and EC with filipin. The three sections in each panel are the phase contrast image (left) and images with (middle) or without (right) the channel for propidium iodide (in red) to show nuclei. **A**, control for staining of UC (no treatment with filipin). **B**, staining of UC with filipin (in cyan). **C**, control for staining of EC (extracted with ethanol but not treated with cholesterol esterase or filipin). **D**, control for completeness of UC removal (extracted with ethanol and treated with filipin but not cholesterol esterase). **E**, staining of EC (extracted with ethanol and sequentially treated with cholesterol esterase and filipin). Exposure time in panels A and B was 15 msec and that in panels C–E was 400 msec. Faint fluorescence in panel C with no filipin treatment is due to increased exposure time as compared to panel A leading to detection of autofluorescence from the RPE. Fluorescence is not increased in panel D indicating complete removal of UC, yet is more pronounced in panel E indicating that EC is mainly present in BrM.

Similar to earlier histochemistry studies [50], [51], UC was broadly distributed in all layers of human NR with only the OS cholesterol content being below the limits of detection by filipin staining (Fig. 8B). The latter is consistent with the much higher cholesterol levels at the base of the OS, bordering the IS, than at the distal tip of the OS, facing the RPE [52], [53]. In the NFL/GCL, IPL, OPL, and IS, both plasma membranes and cell interiors were fluorescent, whereas in the INL and ONL, mainly plasma membranes appeared stained because perikarya of those cells are small. The RPE and BrM contained UC as well [22]. In contrast, the levels of EC were very low/below the limits of detection. This form of cholesterol seemed to be mainly associated with BrM (Fig. 8E), consistent with previous descriptions [22], [26]. To detect fluorescence from EC, image acquisition time was increased ∼25-fold relative to imaging of sections stained for UC (Fig. 8B), consistent with low EC in peripheral BrM relative to macula. This increased exposure also captured bis-retinoid-mediated autofluorescence from the RPE (compare Fig. 8A and 9C). Our data are in agreement with previous results demonstrating that cholesterol is present almost exclusively as UC in the NR [20], [49], [54], and as a mixture of UC and EC in BrM [22]. Absence of significant filipin staining of the OS is consistent with our IH studies indicating that OS do not express principal proteins required for cholesterol synthesis, uptake, elimination and regulation.

## Discussion

By utilizing the PCR array and investigating key proteins of cholesterol synthesis, uptake, efflux, catabolism and regulation, the present work focused on overall retinal cholesterol homeostasis rather than its one specific aspect. Our studies conducted on human specimens complement those done on rodents, a popular model that differs significantly from humans in key aspects of cholesterol and lipoprotein metabolism [3], [55]. Multiple donors were analyzed, thus enabling assessment of inter-individual variability. This work is a part of a larger study by this laboratory in which the NR and RPE from the same donors are comprehensively characterized by different methods [20], [56], [57].

The analysis by the PCR array (Fig. 3) showed that the NR and RPE express most of the genes necessary for cholesterol homeostasis, in agreement with previous findings that the retina can synthesize cholesterol endogenously [58], [59] and also expresses proteins that mediate cholesterol transport [16], [21], [60] and enzymatic removal [20], [42], [43], [44], [56]. Detection of many cholesterol-related genes suggests that cholesterol homeostasis in the NR and RPE could be relatively independent from the rest of the body, consistent with the presence of the blood-retina barrier. However, the extent of this autonomy remains to be determined; we only know that the NR and RPE, which forms a part of the blood-retina barrier, acquire cholesterol from LDL and HDL in the systemic circulation [14], [15], yet the ratio between blood-borne cholesterol and that synthesized in the retina is currently unknown.

Important insights were also obtained from studies by qRT-PCR. These measurements confirmed the expression of 16 genes that we selected for characterization and enabled a comparison of the mRNA levels with the protein levels of CYPs 27A1 and 46A1 determined previously by us by mass spectrometry [56], [57]. In the NR, similar gene expression of each CYP27A1 and CYP46A1 in donors 12 and 13 correlated well with similar protein amounts [56]. Yet, in the RPE, a 13- and 15-fold, respectively, higher mRNA levels for CYPs 27A1 and 46A1 in donor 13 *vs.* donor 12 corresponded only to a ∼1.4- and 1.5-fold increase in protein [57]. In contrast, in the brain, the message levels for each CYP27A1 and CYP46A1 were similar in donors 1–4, consistent with similar protein concentrations [56]. The mean cerebral mRNA content for CYP27A1 was ∼8-fold lower than in the NR (Fig. 5), in a good agreement with protein quantifications also showing much lower (∼5-fold) enzyme levels in the brain [56]. The data obtained demonstrate that in general but not always mRNA levels are a good predictor of protein expression. Therefore mRNA expression should always be validated by other methods.

Of interest is the finding that the variations in gene expression are higher in the RPE than in the NR (Figs. 4–6). This could be due to the “gate-keeping” function of the RPE to control cholesterol and nutrient flux from systemic circulation to the NR and reverse transport of metabolites from the NR back to systemic circulation. Indeed, as a gate-keeper, the RPE has to quickly adjust its gene expression in response to constant fluctuations in the blood content, and in different individuals this adjustment will be different and depend on the blood lipid profile, health status, age, gender, lifestyle, diet and genetic background. Accordingly, in the RPE the scavenger receptor CD36 showed the highest inter-individual variability in gene expression (∼23-fold, Fig. 6) followed by a lower variability in expression of genes for enzymatic cholesterol removal (CYPs 27A1 and 46A1, ∼16-fold, Fig. 5), regulation (LXRβ, ∼12-fold, and SREBP2, ∼7-fold, Fig. 4), efflux (ABCA1, ∼5-fold, Fig. 4), and endogenous cholesterol synthesis (HMGCR, ∼4-fold, Fig. 4).

Studies by IH were conducted to evaluate protein expression of the genes detected by qRT-PCR. HMGCR, LDLR, ABCA1, CYPs 27A1, 46A1 and 11A1 have already been immunolocalized in the retina by others but in species other than humans [13], [14], [16], [42], [43], [44], [45], [46]; immunostainings of SREBPs, SCAP, LXRs and Insigs were novel. Within the NR, immunoreactivity of the studied proteins was confined to specific layers, suggesting that localization to cellular or sub-cellular compartments will eventually be possible. Labeling was not obviously localized to radial fibers evocative of Müller cells, suggesting that either these cells are not labeled along their entire length or that neurons express the studied proteins as well as these retina-specific radial glia. Future double-labeling studies with cell-type specific markers will settle these questions. Compared to previous IH localizations [13], [14], [16], [42], [43], [44], [45], [46], staining patterns in humans were more widely distributed than previously seen in other species, even when the same Abs were used. In particular, immunoreactivity for LDLR was not confined (as in the case with some plasma membrane proteins [61]) to apical or basolateral domains of RPE nor was it near blood vessels, where uptake from systemic circulation might be the principal function. This suggests additional functions for this receptor, perhaps involvement in the intra-retinal transport of lipoproteins as postulated by others [16], [62]. More work is required to determine the basis of inter-study variations in IH stainings.

IH localizations by us and others, however, were consistent in revealing a layer in the NR, the OS, that had weak or absent signal for most of the studied proteins and also for cholesterol as assessed by staining with filipin. Cholesterol, however, is present in this layer as shown by a more sensitive enzyme assay [52], [53]). Low cholesterol content and apparent lack of the key proteins involved in cholesterol biosynthesis, uptake, metabolisms, efflux and regulation suggest that the OS are very different in terms of cholesterol maintenance as compared to other retinal layers. Indeed, low or absent expression and regulation of HMGCR and LDLR in the OS point to alternate mechanism(s) of cholesterol input, perhaps intracellular transport from the IS to OS. This transport could involve a known intracellular cholesterol transporter NPC1L1-like protein which is present in many cells including retinal, and whose deficiency results in striking retinal degeneration also involving degeneration of the OS [39], [63]). Besides transport, the IS could also provide cholesterol for the OS via passive diffusion because IS have a higher cholesterol content than OS. This would explain cholesterol gradient in the OS with the highest sterol concentration in the region bordering the IS [52], [53], [64].

Cholesterol removal from the OS also seems to rely on mechanism(s) other than ubiquitous ABCA1-mediated efflux and metabolism to oxysterols by cholesterol-catabolizing CYPs as these proteins (except CYP46A1) are not present in the OS (Figs. 7,8). These alternate mechanisms could be the calveolin-dependent pathway [65] and/or passive diffusion [66]. Passive diffusion is driven by the gradient and is known to be enhanced by: 1) cholesterol esterification outside the cell by LCAT; 2) plasma membrane receptor SR-BI, which tethers lipoproteins to the cell surface and induces cholesterol redistribution in plasma membranes, and 3) interaction with extracellular HDL [66]. Both LCAT, SR-BI and apoA1, the main protein of HDL, were shown by IH to be expressed in the OS in monkey retina, and the OS-associated immunoreactivity for apoA1 and LCAT was proposed to reflect localization of these proteins within the interphotoreceptor matrix [16]. While it was suggested that the OS acquire lipids from the HDL-like particles [16], we propose that the OS also offload cholesterol to these particles. Indeed, SR-BI is known to mediate bi-directional cholesterol flux between cells and lipoproteins with the direction of the flux depending on the direction of the cholesterol gradient: inside the cell upon interaction with cholesterol-rich mature HDL and outside the cell if cholesterol-poor nascent HDL bind [67], [68]. We propose that the net result of the SR-BI-mediated flux in the OS is cholesterol offload rather than cholesterol supply. This offload would minimize daily retinal cholesterol loss from phagocytosis and, thus, the amount of cholesterol that has to be replenished either via endogenous biosynthesis and/or cholesterol delivery from systemic circulation, both energy-consuming processes [69], [70]. Also, cholesterol offload would be in agreement with experimental data showing that high-cholesterol environment in the basal OS disks reduces the efficiency of the phototransduction cascade [64], the key event in the vision process.

Immunolocalizations also provided important insight pertaining to the RPE. The RPE contained only very faint fluorescence for SREBPs and LXRα (Fig. 7) suggesting weak to absent SREBP regulation of cholesterol biosynthesis and LDL uptake. Apparent lack of SREBPs indicates that: 1) other mechanisms (e.g., HMGCR protein degradation via sterol-accelerated ubiquitination or inhibition by phosphorylation [69], [71]) possibly control cholesterol input to RPE; 2) cholesterol homeostasis in the RPE is regulated, at least in part, by LXRβ at the level of cholesterol output; and 3) cholesterol input to the RPE is likely poorly controlled. The latter is consistent with previous *in vivo* investigation in rats suggesting constant, unregulated uptake of blood-borne LDL by the retina [14], and with cell culture studies showing internalization of large amounts of LDL by the human-derived RPE cells ARPE19 [72]. If indeed true, weak regulation of cholesterol input in the RPE could be one of the factors underlying the development of AMD.

In the RPE, cholesterol could be directed into several different pathways as suggested by available experimental evidence: 1) be esterified and form a complex with apoB-containing particles which are excreted through the basal side of the RPE to BrM and then to the circulation [21], [60]; 2) be assembled into apoA1- and apoE-containing HDL-like particles and transported by ABCA1 through the apical side of the RPE into the interphotoreceptor matrix [16]; 3) be converted to more secretable oxysterols by CYPs [20] that quickly leave the cell and become associated with circulating HDL or albumin [73], [74]; and 4) be esterified and stored in lipid droplets that occasionally appear in the RPE [21]. Of these pathways, removal via the apoB-mediated transport is suggested to play an important role in the pathogenesis of AMD, a devastating blinding disease in elderly. The apoB-containing particles accumulate with age in BrM and form deposits rich in EC and UC called drusen, a hallmark of AMD [21], [60]. Factors affecting lipid deposition in BrM are under investigation [21], [60] but not fully understood. One of them could be the intensity of apoB particle secretion by the RPE in BrM which in turn depends in part on the amount of cholesterol that needs to be eliminated at a given time, in addition to the amount of fatty acids available for esterification to cholesterol. The present study demonstrates that in the RPE, the mRNA levels of the key proteins controlling pathways of cholesterol output and input (HMGCR, LDLR, LXRβ, ABCA1, SREBPs and CYPs) vary significantly between individuals, and that the SREBP regulation is weak. Hence, it is possible that in some individuals there is an imbalance in protein expression of HMGCR and LDLR determining cholesterol input, and ABCA1 and CYPs mediating cholesterol removal leading to increased RPE cholesterol levels. If this is the case, apoB-particle secretion would be increased, and more lipids would be trapped in BrM with age, thereby increasing predisposition to AMD. Further studies are needed to test this notion. While likely important, inter-individual variability in expression of cholesterol-related genes and their weak transcriptional regulation in the RPE by no means are the only factors that probably determine susceptibility to AMD; gene variants are important as well. Two recent genome wide scans identified HDL-related genes (hepatic triglyceride lipase, CETP, ABCA1 and lipoprotein lipase) as risk factors for AMD [17], [18]. However, due to expression of LIPC and CETP in the NR, and the fact that the different single nucleotide polymorphisms have opposite effects on plasma HDL, it is not clear that the effect of these genes is on plasma HDL, or on intra-retinal pathways, or both. Besides the HDL-related genes, CYP27A1 could also be involved in AMD, because its deficiency in humans leads to premature retinal senescence with drusen and changes in RPE [75].

In summary, the present study examined the largest number of cholesterol-related genes and proteins in the retina and RPE and provided novel important insights into cholesterol maintenance of this important tissue.

## Supporting Information

Figure S1 **Quality of CYP27A1 and CYP46A1 Abs.** **A**, Western blot analysis of the homogenate prepared from human NR with Abs against CYP27A1. **B**, IH localizations of CYP46A1 in knockout (CYP46A1-/-) and wild type mice using primary Abs at dilutions identical to those employed for IH of human retinas. Nuclei were stained by DAPI (blue) and immunoreactivity was detected by DyLight 649 conjugated secondary Abs (red). Staining with per-immune serum served as a negative control. Scale bars and abbreviations of retinal layers are the same as in Figs. 7 and 2, respectively.
(DOCX)

Text S1 Full names of the genes investigated in the present work.
(DOCX)

Table S1 Information on the donors whose tissues were used in the present study.
(DOCX)

Table S2 Primers for qRT-PCR.
(DOCX)

Table S3 Primary antibodies tested in the present study.
(DOCX)
