# Supplementary material for: Spatial Distribution of the Pathways of Cholesterol Homeostasis in Human Retina
Source: PLoS One. 2012 May 22;7(5):e37926. doi: 10.1371/journal.pone.0037926 (PMC3358296; doi:10.1371/journal.pone.0037926)
Supplement: Figure S1 — Quality of CYP27A1 and CYP46A1 Abs. A, Western blot analysis of the homogenate prepared from human NR with Abs against CYP27A1. B, IH localizations of CYP46A1 in knockout (CYP46A1-/-) and wild type mice using primary Abs at dilutions identical to those employed for IH of human retinas. Nuclei were stained by DAPI (blue) and immunoreactivity was detected by DyLight 649 conjugated secondary Abs (red). Staining with per-immune serum served as a negative control. Scale bars and abbreviations of retinal layers are the same as in Figs. 7 and 2, respectively. (DOCX) [file pone.0037926.s001.docx]

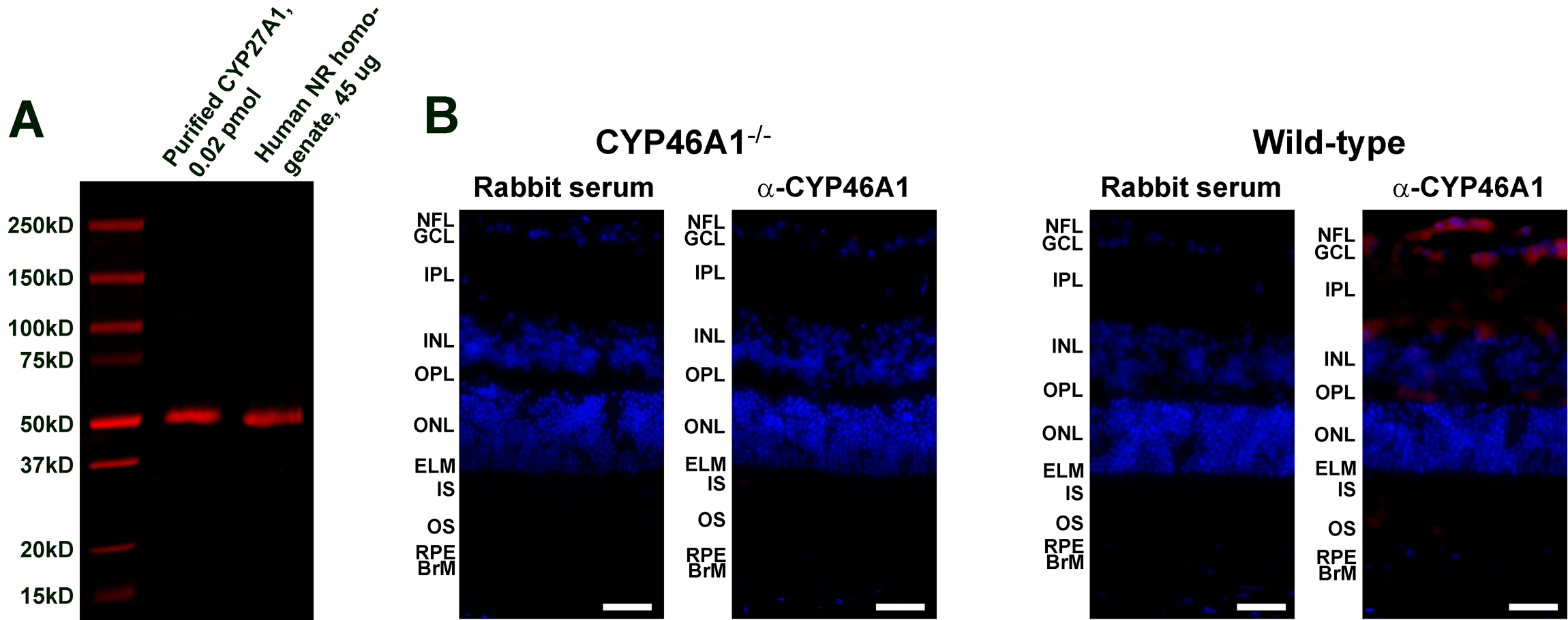


**Figure S1.** **Quality of CYP27A1 and CYP46A1 Abs. A**, Western blot analysis of the homogenate prepared from human NR with Abs against CYP27A1. **B**, IH localizations of CYP46A1 in knockout (CYP46A1^-/-^) and wild type mice using primary Abs at dilutions identical to those employed for IH of human retinas. Nuclei were stained by DAPI (blue) and immunoreactivity was detected by DyLight 649 conjugated secondary Abs (red). Staining with per-immune serum served as a negative control. Scale bars and abbreviations of retinal layers are the same as in Figs. 7 and 2, respectively.
