# Supplementary material for: Spatial Distribution of the Pathways of Cholesterol Homeostasis in Human Retina
Source: PLoS One. 2012 May 22;7(5):e37926. doi: 10.1371/journal.pone.0037926 (PMC3358296; doi:10.1371/journal.pone.0037926)
Supplement: Text S1 — Full names of the genes investigated in the present work. (DOCX) [file pone.0037926.s002.docx]

**Text S1.** Full names of the genes investigated in the present work

ABCA1, ATP-binding cassette transporter A1; ABCG1, ATP-binding cassette transporter G1; ACAA2, Acetyl-Coenzyme A acyltransferase 2; ANGPTL3, Angiopoietin-like 3; ANKRA2, Ankyrin repeat A2; APOA4, Apolipoprotein A-IV; APOD, Apolipoprotein D; APOE, Apolipoprotein E; APOL1, Apolipoprotein L, 1; APOL2, Apolipoprotein L, 2; CDH13, Cadherin 13; CEL, Carboxyl ester lipase; CNBP, CCHC-type zinc finger nucleic acid binding protein; COLEC12, Collectin 12; CXCL16, Chemokine (C-X-C motif) ligand 16; CYB5R3, Cytochrome b5 reductase 3; CYP11A1, Cytochrome P450 11A1; CYP39A1, Cytochrome P450 39A1; CYP46A1, Cytochrome P450 46A1; CYP51A1, Cytochrome P450 51A1; CYP7B1, Cytochrome P450 7B1; DHCR24, 24-dehydrocholesterol reductase; DHCR7, 7-dehydrocholesterol reductase; ELA3A, Elastase 3A; ELA3B, Elastase 3B; FDFT1, Farnesyl-diphosphate farnesyltransferase 1; FDPS, Farnesyl diphosphate synthase; HDLBP, High density lipoprotein binding protein; HMGCR, 3-hydroxy-3-methylglutaryl-Coenzyme A reductase; HMGCS1, 3-hydroxy-3-methylglutaryl-Coenzyme A synthase 1; HMGCS2, 3-hydroxy-3-methylglutaryl-Coenzyme A synthase 2; IDI1, Isopentenyl-diphosphate delta isomerase 1; IDI2, Isopentenyl-diphosphate delta isomerase 2; Insig1, Insulin induced gene 1; Insig2, Insulin induced gene 2; LCAT, Lecithin-cholesterol acyltransferase; LDLR, Low density lipoprotein receptor; LDLRAP1, LDLR adaptor protein 1; LRP10, LDLR-related protein 10; LRP12, LDL-related protein 12; LRP1B, LDL-related protein 1B; LRP6, LDLR-related protein 6; LRPAP1, LDLR-related protein associated protein 1; MBTPS1, Membrane-bound transcription factor peptidase, site 1; MVD, Mevalonate decarboxylase; MVK, Mevalonate kinase; NSDHL, NAD(P) dependent steroid dehydrogenase-like; OLR1, Oxidized LDLR 1; OSBPL1A, Oxysterol binding protein-like 1A; OSBPL5, Oxysterol binding protein-like 5; PMVK, Phosphomevalonate kinase; PPARD, Peroxisome proliferator-activated receptor delta; PRKAA1, Protein kinase, AMP-activated, alpha 1 catalytic subunit; PRKAA2, Protein kinase, AMP-activated, alpha 2 catalytic subunit; PRKAG2, Protein kinase, AMP-activated, gamma 2 non-catalytic subunit; SCAP, SREBP chaperone; SCARF1, Scavenger receptor class F1; SNX17, Sorting nexin 17; SOAT1, Sterol O-acyltransferase 1; SORL1, Sortilin-related receptor; SR-BI, scavenger receptor class B, type I; SR-BII, scavenger receptor class B, type II; SREBP1, Sterol regulatory element binding protein 1; SREBP2, Sterol regulatory element binding protein 2; STAB1, Stabilin 1; STARD3, StAR-related lipid transfer (START) domain containing 3; TM7SF2, Transmembrane 7 superfamily member 2; TRERF1, Transcriptional regulating factor 1; VLDLR, Very low density lipoprotein receptor; ZMYND15, Zinc finger MYND-type containing 15.
