# Supplementary material for: Spatial Distribution of the Pathways of Cholesterol Homeostasis in Human Retina
Source: PLoS One. 2012 May 22;7(5):e37926. doi: 10.1371/journal.pone.0037926 (PMC3358296; doi:10.1371/journal.pone.0037926)
Supplement: Table S1 — Information on the donors whose tissues were used in the present study. (DOCX) [file pone.0037926.s003.docx]

**Table S1**. Information on the donors whose tissues were used in the present study

| Donor ID | Age (y) | Gender | Death-to-preservation* (hrs) | Tissue | Past Medical History |
| --- | --- | --- | --- | --- | --- |
| 1 | 50 | M | 6 | Brain, left temporal lobe tip,  gray matter | Schizophrenia, hypertension, type 2 diabetes, severe coronary artery disease, lupus erythematosus; no lipid-lowering treatments. |
| 2 | 87 | M | 11 | Brain, right temporal lobe tip, gray matter | Hypertension, severe coronary artery disease; no lipid-lowering treatments, metastatic carcinoma, no history of dementia, yet the donor had neurofibrillary tangles and senile plaques in the CA1 region of hippocampus but no tangles and plaques in the temporal cortex. |
| 3 | 69 | M | 9 | Brain, left temporal lobe tip, gray matter | Hypertension, hyperlipidemia, statin treatment, severe coronary artery disease; type 2 diabetes, no history of Alzheimer’s disease. |
| 4 | 75 | F | 11 | Brain, left temporal lobe tip, gray matter | Hypertension, atherosclerosis, coronary artery disease with bypass, diabetes type II, rheumatoid arthritis, normal brain, no changes indicative of Alzheimer’s disease. |
| PM008 | 58 | M | 8 | Retina and RPE | Multiple respiratory illnesses (chronic obstructive pulmonary disease and pulmonary fibrosis), anxiety disorder. |
| PM009 | 56 | M | 7 | Retina and RPE | Hypertension, alcohol and tobacco use. |
| PM010 | 61 | M | 10 | Retina and RPE | Alcohol and tobacco use. |
| PM011 | 61 | M | 8 | Retina and RPE | End-stage chronic obstructive pulmonary disease, coronary artery disease. |
| PM012 | 58 | M | 10 | Retina and RPE | Hypertension, chronic obstructive pulmonary disease, Budd-Chiari syndrome, EtOH and tobacco abuse with end-stage liver disease. |
| PM013 | 66 | F | 12 | Retina and RPE | Hypertension, coronary artery disease, hypothyroidism, hypercholesterolemia. |
| PM023 | 67 | M | 10 | Retina and RPE | Hypertension, cardiovascular disease, prostate cancer, anti-hypertensive and cholesterol-lowering medications, tobacco use, EtOH occasionally. |

*Time when eyeballs and processed tissue were placed in paraformaldehyde and -80^o^C freezer,

respectively.
