# Supplementary material for: Spatial Distribution of the Pathways of Cholesterol Homeostasis in Human Retina
Source: PLoS One. 2012 May 22;7(5):e37926. doi: 10.1371/journal.pone.0037926 (PMC3358296; doi:10.1371/journal.pone.0037926)
Supplement: Table S2 — Primers for qRT-PCR. (DOCX) [file pone.0037926.s004.docx]

**Table S2**. Primers for qRT-PCR

| **Gene** | **Primers** |
| --- | --- |
| **ABCA4** | Forward: 5’-AACCAGATCACCGCATTCCT  Reverse: 5’-GGCTGGTTTCAATGTCCCTTC |
| **RPE65** | Forward: 5’-GCCGCTCACAGCTCATGTAA  Reverse: 5’- TGGCTCAGATCCAACTTCAAAG |
| **CYP27A1** | Forward: 5’-GGCAACGGAGCTTAGAGGAGAT  Reverse: 5’-GCCTTGAACGAACAGCTGAAA |
| **CYP46A1** | Forward: 5’-CCTTCTT CATTGCTGGTCACG  Reverse: 5’-TCCATCACTGTGAACGCCAAG |
| **CYP11A1** | Forward: 5’-GAACTTTTTGCCCCTGTTGGAT  Reverse: 5’-TGTGCAGGACACTGACGAAGTC |
| **SREBP1** | Forward: 5'-CGCAAGGCCATCGACTACATT  Reverse: 5'-TGCGCAGACTTAGGTTCTCCTG |
| **SREBP2** | Forward: 5’-ATCGCTCCTCCATCAATGACA  Reverse: 5’-CTTGTGCATCTTGGCGTCTGT |
| **SCAP** | Forward: 5’-TGGCCAGTGGAGGACAAGAT  Reverse: 5’-CCACGGTGAGCAAACACATG |
| **Insig1** | Forward: 5'-TCAACCTGCTGCAGATCCAGA  Reverse: 5'-GGAAAAGATGGTGGCGATCA |
| **Insig2** | Forward: 5’-TTGTCTCTCACACTGGCTGCA  Reverse: 5’-TCCAAGGCCAAAACCACTTCT |
| **HMGCR** | Forward: 5’-GGTGTTCAAGGAGCATGCAAA  Reverse: 5’-AGCCATTACGGTCCCACACA |
| **LXRα** | Forward: 5’-CCCCATGACCGACTGATGTT  Reverse: 5’-CGGAGGCTCACCAGTTTCATT |
| **LXRβ** | Forward: 5’-TCCAGCTAACAGCGGCTCAA  Reverse: 5’-CGGAGAAGGAGCGTTTGTTG |
| **ABCA1** | Forward: 5’-TCTTCCCACATTTTTGCCTGG  Reverse: 5’-CGATTCTCCCCAAACCTTTCC |
| **LDLR** | Forward: 5'-TGAAGTTGGCTGCGTTAATGTG  Reverse: 5'-ATTCGCCGCTGTGACACTTG |
| **SR-BI** | Forward: 5’-TCCA TCTACCCACCCAACGA  Reverse: 5’-TGCAGGTGCTGACGTTCTGA |
| **SR-BII** | Forward: 5’-GGACTGCCTGCCTTTCGGTATA  Reverse: 5’-CGGCATTGTCTGACGTATTGG |
| **CD36** | Forward: 5’-AGGACGCTGAGGACAACACAGT  Reverse: 5’-AAGGTTCGAAGATGGCACCA |
| **β-Actin** | Forward: 5’-ACTGGAACGGTGAAGGTGACA  Reverse: 5’-TCGGCCACATTGTGAACTTTG |
