# Supplementary material for: Spatial Distribution of the Pathways of Cholesterol Homeostasis in Human Retina
Source: PLoS One. 2012 May 22;7(5):e37926. doi: 10.1371/journal.pone.0037926 (PMC3358296; doi:10.1371/journal.pone.0037926)
Supplement: Table S3 — Primary antibodies tested in the present study. (DOCX) [file pone.0037926.s005.docx]

**Table S3**. Primary antibodies tested in the present study

| **Antigen** | **Source** | **Species** | **Clonality** | **Dilution** | **Immunization antigen** |
| --- | --- | --- | --- | --- | --- |
| SREBP1 | **Santa Cruz*** **Biotechnology** | Rabbit | Polyclonal | 1:500 | A peptide within an internal region of human SREBP1 |
| SREBP2 | **Abcam** | Rabbit | Polyclonal | 1:500 | Amino acids 455-469 of human SREBP2 |
|  | Santa Cruz Biotechnology | Rabbit | Polyclonal | 1:50 | Amino acids 812-975 of human SREBP2 |
| SCAP | **Sigma-Aldrich** | Rabbit | Polyclonal | 1:5 | Amino acids 990-1108 of human SCAP |
|  | Santa Cruz Biotechnology | Rabbit | Polyclonal | 1:50 | Amino acids 979-1278 from the C-terminal cytoplasmic domain of human SCAP |
| Insig1 | Santa Cruz Biotechnology | Rabbit | Polyclonal | 1:150 | Amino acids 31-100 near the N-terminus of human Insig 1 |
|  | **Santa Cruz** **Biotechnology** | Rabbit | Polyclonal | 1:50 | A peptide near the N-terminus of human Insig 1 |
| Insig2 | **Santa Cruz** **Biotechnology** | Rabbit | Polyclonal | 1:50 | Amino acids 1-40 of human Insig 2 |
| HMGCR | **United States Biological** | Rabbit | Polyclonal | 1:50 | Amino acids 827-840 of human HMGCR |
|  | Santa Cruz Biotechnology | Rabbit | Polyclonal | 1:250 | Amino acids 589-888 from the C-terminus of human HMGCR |
| ABCA1 | **Abcam** | Rabbit | Polyclonal | 1:1000 | Amino acids 1200-1300 of human ABCA1 |
|  | Lifespan Biosciences | Rabbit | Polyclonal | 1:1000 | Amino acids 1100-1300 of human ABCA1 |
|  | Santa Cruz Biotechnology | Rabbit | Polyclonal | 1:100 | Amino acids 91-310 of human ABCA1 |
| LXRα | **Santa Cruz** **Biotechnology** | Goat | Polyclonal | 1:50 | A peptide near the N-terminus of human LXRα |
| LXRβ | **Santa Cruz** **Biotechnology** | Goat | Polyclonal | 1:150 | A peptide near the C-terminus of mouse LXRβ |
| LDLR | **Abcam** | Rabbit | Monoclonal | 1:250 | A peptide from the C-terminus of human LDLR |
|  | Santa Cruz Biotechnology | Rabbit | Polyclonal | 1:150 | Amino acids 701-820 near the C-terminus of human LDLR |
| CYP27A1 | **Pikuleva’s laboratory** | Rabbit | Polyclonal | 1:2500 | Full-length human CYP27A1 |
| CYP46A1 | **Pikuleva’s laboratory** | Rabbit | Polyclonal | 1:1000 | Full-length human CYP27A1 |
| CYP11A1 | **Pikuleva’s laboratory** | Rabbit | Polyclonal | 1:1000 | Full-length bovine CYP11A1 |

*Abs in bold were used for immunostainings in Figs. 7 and 8.
